# Advanced Field-Effect Sensors

**DOI:** 10.3390/s23094554

**Published:** 2023-05-08

**Authors:** Antonio Di Bartolomeo

**Affiliations:** Physics Department E.R. Caianiello, University of Salerno, 84084 Fisciano, Salerno, Italy; adibartolomeo@unisa.it

## 1. Introduction

Sensors based on the field-effect principle have been used for more than fifty years in a variety of applications ranging from bio-chemical sensing to radiation detection or environmental parameter monitoring. The basic working principle of field-effect sensors is the same as that of field-effect transistors (FETs) in which the conductance between two electrodes (source and drain) is controlled by the electric field generated by a gate [1]. In these sensors, the gate often includes or is connected to the sensing element. 

Field-effect biochemical sensors have found increasing applications for pH and molecular or DNA sensing since the proposal of the ion-sensitive field-effect transistor (ISFET) by Bergveld in 1970 [2,3].

Field-effect devices have been extensively exploited for gas sensing, and photo-activated FETs (Photo-FETs) are actively progressing as chemical and gas detectors. 

Both junction (JFET) and metal–oxide–semiconductor (MOSFET) FETs are widely used as photodetectors and ionizing radiation detectors or dosimeters in radioprotection, radiotherapy, medicine, and dentistry [4,5]. Furthermore, FETs enable sensitive temperature sensors and piezoelectric strain gauges [6].

In the past three decades, the advent of nanostructured materials, either one-dimensional (1D) or two-dimensional (2D), has created opportunities to integrate new sensing materials or to develop innovative architectures in field-effect-based sensors [7,8]. The optimization of existing devices, the experimentation of new field-effect structures and fabrication techniques, and the design of novel electronic systems for signal amplification and processing are currently underway [9].

A great advantage of field-effect sensors is that they provide intrinsic signal amplification and can be integrated with the electronics needed for the sensor’s signal processing on the same semiconductor chip. Moreover, field-effect sensors feature a high sensitivity, low cost, and miniaturization.

Field-effect-based sensing offers several challenges arising from the highly interdisciplinary nature of the applications in which knowledge of material science, surface chemistry and physics, biomolecular kinetics, electronic engineering, etc., is required.

This Special Issue summarizes the recent trends in the research on the fabrication, design, understanding, simulation, and utilization of field-effect sensors, with particular attention for biological and chemical sensing.

## 2. Overview of Contributions

Field-effect sensors based on 1D or 2D nanomaterials have attracted significant attention for biological and chemical sensing. Detailed modeling able to predict the electrical response to different molecules of devices based on a variety of semiconductors is an important prerequisite for the development of performant field-effect sensors and for their integration at the circuit level. For this purpose, Pasadas and coworkers develop a compact model that predicts the electrical read-out of field-effect biosensors based on 2D semiconductors [10]. They consider electrolyte–insulator–semiconductor field-effect biosensors in which a charged, ion-permeable membrane represents the macromolecules formed by the receptors and targets attached to the insulator surface. The model includes the effects of the site-binding charge, the electrolyte screening, and the biomolecule charges at the electrolyte side. The successful validation of the model is achieved by comparing the prediction with the experimental data provided by a MoS_2_-biosensor exposed to variable pH levels and streptavidin concentrations.

Food contamination is an urgent global issue. Among other contaminants, aflatoxins A1 to G1 have a high level of toxicity, yet the current biosensing detection techniques prevent their pervasive monitoring throughout the agri-food chain. To fill this gap, Mo and coworkers propose a pyrrole-based molecular field-effect transistor (MolFET) as a single-molecule sensor for the amperometric detection of aflatoxins [11]. The device utilizes a single polymeric chain composed of a sequence of eight pyrrole monomers as the channel and detection element, and Au is used as the source and drain. Ab initio atomistic calculations are applied to show that the poly-pyrrole chain can detect a single aflatoxin B1 at a time and achieve the ultimate potential sensitivity at the single-molecule level. Moreover, it is shown that a gate terminal can be the key element to enhance the sensitivity of the aflatoxin B1 single-molecule sensor.

Derived from the ISFET, the extended-gate field-effect transistor (EGFET) is an electrochemical biosensor that is used to measure ion concentrations in solutions. Like the ISFET, it can be applied for pH sensing, DNA detection, glucose monitoring, etc. While in an ISFET, the metal gate of the MOSFET is replaced by an ion-sensing film, an electrolyte solution, and a reference electrode, the EGFET retains the complete MOSFET structure, but the gate is extended for the connection to the metal-sensing film electrolyte solution and the reference electrode. Kuo and coworkers use CMOS technology to fabricate a biosensor for the detection of acid lactic [12]. The sensor uses an EGFET with a sensing window on an extended gate composed of a RuO_2_ film sputtered on six metal layers and functionalized with lactase. To ease the detection of lactic acid, a microfluidic system is integrated with the biosensor. The EGFET biosensor is tested experimentally, confirming its good sensitivity and linearity.

A similar application is presented by Schuck and coworkers, who propose a graphene-based lactate biosensor to detect the concentrations of L-lactic acid in a buffer solution and blood plasma [13]. To guarantee its stability and increase its selectivity, the graphene active surface is functionalized with the lactate dehydrogenase enzyme using different substances, such as chitosan, glutaraldehyde, and nafion. The transfer characteristics of the graphene in the FET structure are measured with different concentrations of L-lactic acid. The results show that the device demonstrates stable operation for several days and a linear response for a wide range of concentrations.

Aiming for easier integration with lab-on-a-chip devices, Panahi and collaborators develop a FET biosensor called an open-gate junction field-effect transistor (OG-JFET) for biomolecular to biological cell analyses [14]. The OG-JFET consists of a p-type channel on top of an n-type layer in which the p-type channel serves as the sensing conductive layer between two ohmic contacted sources and drain electrodes. The reference electrode in the solution is eliminated, and a back gate controls the channel conduction. The sensor is operated at a low voltage. The OG-JFET is tested with a pH solution, human exhalation vapor, oral cell neutrophils, and ssDNA molecules, which are efficiently detected as variation in the source–drain current.

S.K. Cho and W.J. Cho [15] propose an enzymatic field-effect transistor (EnFET) for urea detection that is based on an amorphous indium gallium zinc oxide (a-IGZO) thin-film transistor. The EnFET sensor consists of a SnO_2_ extended gate with immobilized urease as the sensing unit and a triple-gate a-IGZO transistor as the transducer unit. The extended gate is separated from the transducer such that the low-cost extended gate can easily be exchanged, and the expensive FET transducer can be continuously used without damage. The device exhibits a high sensitivity for urea detection and is promising for the sensing of DNA, enzymes, antigen antibodies, cells, and hormones.

Within the ISFET sensor family, water-gate thin-film transistors (WGTFT) [16], which incorporate an electric double layer capacitor into the transistor itself and use the aqueous sample as an active part of the field-effect device, have been widely used for the detection of aqueous analytes such as organic pollutants, ions, viruses, and hormones. AlQahtani and coworkers present a WGTFT biosensor with a parallel potentiometric and capacitive response that utilizes SnO_2_ as the semiconductor, chosen for its good stability and immunity to electrochemical doping [17]. The response is based on the analysis of the output characteristics rather than the transfer characteristics. To satisfy a recent compelling need, it is shown that the device can detect the antibody (immunoglobuline IgG1) against the SARS-CoV-2 virus.

SnO_2_ thin films have often been exploited in oxygen gas sensors. However, their ability to detect dissolved oxygen in solutions is still unknown. Hence, Noda and coworkers use an SnO_2_-gate FET to develop a method for investigating the dissolved-oxygen-sensing properties of SnO_2_ thin films in solutions [18]. The transfer characteristics and the sensitivity of the device are experimentally obtained and compared to those of a similar Si_3_N_4_-gate FET. It is shown that only SnO_2_-gate FETs undergo a shift in threshold voltage in response to a decrease in the concentration of dissolved oxygen. As SnO_2_ responds to both hydrogen ions and dissolved oxygen, a pH correction is needed. Since Si_3_N_4_ has ion selectivity only towards hydrogen ions, the correction can be provided by combining the SnO_2_- and Si_3_N_4_-based FETs in a multimodal sensing device. Both SnO_2_- and Si_3_N_4_-based FETs can be integrated into a standard CMOS process, making the multimodal sensing approach easy to implement.

Al-Khalqi and coworkers propose an electrolyte insulator semiconductor (EIS) device for pH sensing that is based on ZnO-nanorod-sensing membrane layers doped with magnesium [19]. The ZnO nanorods, which are prepared via a hydrothermal process and doped with 3% Mg, exhibit a high hydrogen ion sensitivity. Due to their small dimensions, the nanorods are suitable for constructing sensing devices because they easily bind with chemical and biological reagents. The EIS sensor with Mg-doped ZnO nanorods exhibits a detection sensitivity higher that the sensitivity achieved with EIS devices based on ZnO thin films.

The detection of metal ions is an important need as metal ions are essential to the human body and play an important role in human health. Budhi Laksana and coworkers investigate a biosensor using quercetin and copper (Cu^2+^) ions as molecules for detecting the molecular absorption of metal complexes [20]. The proposed biosensor consists of a silicon nanowire FET (bio–NWFET) platform able to detect various concentrations of copper based on quercetin–metal ion interactions. The bio–NWFET is endowed with two gates. The back gate controls the typical transistor behavior, while the front gate is used as a sensing area that is sensitive to physical stimuli. As the quercetin–Cu^2+^ complex exhibits strong absorption at wavelengths of 430–450 nm, the transistor is used as a photodetector (opto-FET) with an absorbance level that depends on the Cu^2+^ concentration at the wavelength of 450 nm. The opto–FET sensor shows the linear and sensitive detection of low Cu^2+^ concentrations.

Chemical gas sensors are omnipresent and of extreme importance in industrial process control, as safety systems in various fields, and for healthcare. Several gases can be very harmful to human health. Among the various detector types, semiconductor-based devices are the most used because of their rapid and efficient gas detection. They are based on a variety of semiconducting materials such as metal oxides, organic materials, perovskites, carbon nanotubes, and two-dimensional materials [21]. Their possible drawbacks are a high reactivity to gases other than the target gas, low recovery rates, and a limited sensitivity to extremely small amounts of gas. As a solution, photo-activated or photo-assisted gas detection with diverse materials and devices has been proposed. Photo-activated gas sensors exploit light of a specific wavelength during the gas detection operation to increase the gas reactivity, enhance the photoreaction and recovery speed and secure gas selectivity. The principle of these devices is based on photo-generated charge carriers that interact with the oxygen ions of the gas, resulting in a change in conductivity. The recent progress in photo-activated gas sensors is summarized in a review paper by Lee and Yoo [22]. The photo-activated gas detectors are classified according to the type of target gas and light source in the ultraviolet or visible range. Particular attention is given to photo-activated gas detectors with a high selectivity for nitric oxide, nitric dioxide, formaldehyde, and ammonia gases.

Domènech-Gil and coworkers deal with the development of a technology for selective gas sensing [23]. Virtual electronic noses are combined with machine learning techniques to discriminate between oxygenated volatile organic compounds in the parts-per-billion concentration range. Their approach is based on the use of one single sensor operating as a virtual sensor array and the use of machine learning algorithms to enhance the selectivity among similar gases. The sensor is a silicon-carbide-based FET endowed with a nanostructured porous iridium gate that promotes the optimal gas sensing mechanisms. The proposed sensor can differentiate between three oxygenated volatile organic compounds (formaldehyde, formic acid, and acetic acid) that appear sequentially as products and by-products of each other and to quantify their concentrations. The combination of the virtual electronic nose coupled with computing power is a viable approach for the development of selective gas-sensor technologies.

The miniaturization of field-effect gas sensor packages is a not trivial problem as the sensor must interact with an external environment. In their work, Samotaev and coworkers promote monolithic sintered ceramics laser processing technology to obtain metal–ceramic cases [24]. A step-by-step strategy for the miniaturization of capacitive field-effect gas sensors is provided. The metal–ceramic package for a fully functional solid-state field-effect semiconductor hydrogen sensor is manufactured by combining various material processing and deposition methods of such as laser micromilling, inkjet printing, and sputtering. As the chip temperature and its fluctuations can affect the gas sensitivity, the package is endowed with a built-in heater, which is necessary for the stabilization of the operating temperature.

Electromagnetic wave imaging is widely exploited in external body detection, microcrack/void detection, and security gate checks. It exploits the transmission and reflection characteristics of wireless signals. To obtain transmitted images of objects on a conveyor belt, a sub-terahertz imaging system utilizes a transmitting block that radiates sub-terahertz signals, optical components to evenly apply the signal to the objects on the belt, and a receiving block that captures the signal intensity. The spatial resolution of the system is determined by the detector element density and the operating frequency. Lee and coworkers introduce a detector array structure that increases the spatial resolution of the sub-terahertz imaging of a target on a conveyor belt [25]. They realize a system consisting of two lines of a detector array that are intentionally misaligned. In their design, the detectors in the second column are placed between those in the first column. The detector module includes an on-chip antenna, a complementary metal–oxide–semiconductor (CMOS) detector core, a preamplifier, and a buffer amplifier. The resolution is essentially related to the size and interval of the detector pixel. The image resolution is improved in the direction perpendicular to the movement of the belt.

## 3. Conclusions

This Special Issue offers a balanced coverage of the contemporary trends in the development of field-effect sensors for biological and chemical sensing.

Several device architectures, namely the EGFET, OG-JFET, WGTFT, EIS, bio–NWFET, and opto–FET, are presented and thoroughly discussed. Field-effect devices for gas sensing are also widely explored, both with traditional and light-assisted approaches.

The Special Issue shows that the research on field-effect sensors is progressing in two main directions. The first direction involves the use of new materials and experimentation with new architectures. The second direction exploits existing devices in intelligent systems that benefit from efficient data analysis or machine learning algorithms. Both research lines are contributing to the development field-effect sensors with ever-increasing sensitivity, durability, and a low cost.
